# Maternal nutrition practices in Uttar Pradesh, India: Role of key influential demand and supply factors

**DOI:** 10.1111/mcn.12839

**Published:** 2019-06-18

**Authors:** Phuong Hong Nguyen, Shivani Kachwaha, Rasmi Avula, Melissa Young, Lan Mai Tran, Sebanti Ghosh, Rajeev Agrawal, Jessica Escobar‐Alegria, Sumeet Patil, Purnima Menon

**Affiliations:** ^1^ Poverty, Health and Nutrition Division International Food Policy Research Institute (IFPRI) Washington DC USA; ^2^ Emory University Atlanta Georgia; ^3^ FHI360 Washington DC; ^4^ Network for Engineering, Economics, Research and Management (NEERMAN) Mumbai India

**Keywords:** calcium, diverse diet, India, iron and folic acid, maternal nutrition

## Abstract

Despite strong policy and program commitment, essential maternal nutrition services are not reaching enough women in many countries. This paper examined multifactorial determinants (personal, family, community, and health services) associated with maternal nutrition practices in Uttar Pradesh, India. Data were from a household survey of pregnant (*n* = 667) and recently delivered women (*n* = 1,835). Multivariable regression analyses were conducted to examine the determinants of four outcomes: consumption of diverse diets, consumption of iron folic acid (IFA) and calcium tablets, and weight monitoring during pregnancy. Population attributable risk analysis was used to estimate how much the outcomes can be improved under optimal program implementation. During pregnancy, women consumed 28 IFA and 8 calcium tablets, 18% consumed diverse diet, and 17% were weighed ≥3 times. Nutrition knowledge was associated with consumption of diverse diet (odds ratio [OR] = 2.2 times), IFA (2.3 times), calcium (11.7 times), and weight monitoring (1.3 times). Beliefs and self‐efficacy were associated with IFA (OR = 2.0) and calcium consumption (OR = 4.6). Family support and adequate health services were also associated with better nutrition practices. Under optimal program implementation, we estimate that 51% of women would have adequate diet diversity, an average consumption of 98 IFA, and 106 calcium tablets, and women would be weighed 4.9 times during pregnancy. Strengthening existing program operations and increasing demand for services has the potential to result in large improvements in maternal nutrition practices from current baseline levels but may not be sufficient to meet World Health Organization‐recommended levels without creating an enabling environment including improvements in education and income levels to support behaviour change.

**Key messages**

- Uttar Pradesh, a densely populated state with high burden of undernutrition, lags behind India in maternal nutrition practices: 18% of pregnant women met recommendation of dietary diversity, women consumed 28 IFA and 8 calcium tablets, and they were weighed only 1.3 times during pregnancy.
- Maternal nutrition practice (consumption of diverse diets, IFA and calcium supplementation, and weight monitoring) was each influenced by differing sets of multilevel determinants (individual, family, community, and health service factors).
- Under optimal program implementation, we estimated that around half of the women would have adequate diet diversity, an average consumption of 98 IFA, and 106 calcium tablets, and women would be weighed 4.9 times during their pregnancy.

## 1 INTRODUCTION

Maternal undernutrition has been a long‐standing public health concern globally due to its adverse consequences on mortality and morbidity burden for mothers and their children (Black et al., 2013). Despite well‐established evidenced‐based interventions, limited progress has been made in reducing maternal undernutrition; especially in South Asia (Bhutta et al., 2013). The prevalence of maternal low‐body‐mass index (BMI <18.5) is high at 10–20% globally, but alarming at 30–40% in South Asia (Black et al., 2008). It is estimated that 38% of pregnant women (PW; ~32 million) globally are anaemic (World Health Organization [WHO], 2011), with the highest incidence and number of anaemia cases in South Asia (Stevens et al., 2013). Based on current global trends, it is estimated that it would take approximately 60 years before anaemia rates would drop to 15%, and in South Asia it would take more than a century (Mason, Martorell, Saldanha, & Shrimpton, 2013). There is a clear need for prioritization of maternal nutrition and improved implementation of existing programs.

India, which carries a third of the burden of undernutrition globally, has particularly disadvantaged maternal endowments at the beginning of pregnancy. Nearly a third of Indian women (International Institute for Population Sciences [IIPS], 2017) and 45% of adolescent girls (RSoC, 2014) have a low BMI and thus risk entering pregnancy in a vulnerable state. Indian women gain only 7‐kg weight, on average, during pregnancy, much lower than the recommended weight gain of 10–12 kg (Coffey, 2015). More than half of pregnant and non‐PW in India are anaemic (IIPS, 2017), and progress to reduce anaemia has been slow in the last decade (Nguyen, Scott, Avula, Tran, & Menon, 2018).

The government of India aims to provide multipronged maternal and child nutrition and health services through the Integrated Child Development Scheme and Reproductive, Maternal, Newborn, Child & Adolescent Health (RMNCH)+A programs. These ambitious programs seek to universally expand the quality and coverage of maternal health services, including iron folic acid (IFA) and calcium supplementation, weighing of PW, and counselling on diet, weight gain, rest, and hygiene. Despite a strong policy commitment and program priority to improve maternal nutrition, many proven maternal nutrition interventions are not yet reaching majority of women during pregnancy. Although nearly 90% of PW in India were registered for health services, only half of them received at least four antenatal care (ANC) visits and only a third of them reported consuming IFA tablets for ≥100 days (Avula et al., 2018). This indicates a considerable gap in access to and utilization of existing maternal nutrition services and calls for special action toward improving program implementation and effectiveness.

The determinants of maternal nutrition practices are complex, and each of the practices may have potential differing set of determinants (Table S1). Few studies have examined the determinants for a diverse set of maternal nutrition practices together, and none have been undertaken in the context of Uttar Pradesh, a state with population over 200 million and annual birth cohort over 5 million. Uttar Pradesh also had a high‐burden of undernutrition and lags behind India in access to and utilization of the maternal nutrition services with only 26% of the women who received at least four ANCs and only 13% of the women consumed IFA tablets for ≥100 days (IIPS, 2016).

Alive and Thrive (A&T) is an initiative that supports scaling up of nutrition interventions to save lives, prevent illnesses, and contribute to healthy growth and development through improved maternal nutrition and infant and young child feeding practices in several countries. To address the challenges of maternal undernutrition in Uttar Pradesh, A&T aims to test the feasibility of improving the provision and uptake of a package of maternal nutrition interventions, which include provision of and counselling on IFA and calcium supplements, adequate weight monitoring during pregnancy and counselling on weight gain, interpersonal counselling on diet during pregnancy and on breastfeeding during postpartum, and community mobilization.

In the context of this implementation research effort, this paper used baseline data to examine how the preintervention maternal, household, community, and health service factors influenced four specific maternal nutrition practices. The findings can help identify key factors, which if strengthened, could markedly improve maternal nutrition practices and outcomes in the context of this program.

## 2 METHODS

### 2.1 Data and study population

Data were obtained from a baseline household survey conducted in 2017 as part of the above‐mentioned maternal nutrition program evaluation in Uttar Pradesh (http://ClinicalTrials.gov Identifier: NCT03378141). The survey was carried out in 26 rural blocks from two districts (Unnao and Kanpur‐Dehat). A sample of 600 PW and 1,800 recently delivered women (RDW) with infants under 6 months of age was selected following a two‐stage cluster sampling: (a) seven Gram Panchayats per block were selected using probability proportional to size and then (b) up to four PWs and 13 RDWs were selected using systematic random sampling. A sample frame of PWs and RDWs was constructed by listing 300–350 households in each Gram Panchayat.

Data were collected using a structured questionnaire administered on tablets by the survey team appointed by Network for Engineering and Economics Research and Management. Survey enumerators were trained through lectures, role‐play, mock‐interviews in classroom settings, and during field practice. Ethical approval was obtained from Suraksha Independent Ethics Committee in India and the International Food Policy Research Institute (USA). Verbal informed consent was obtained from all participants.

### 2.2 Dependent variables

We constructed four primary outcomes related to the four maternal nutrition practices: dietary diversity during pregnancy, consumption of IFA and calcium during last pregnancy, and routine weight monitoring during last pregnancy. Data from PW were used to assess determinants of current diet during pregnancy, and data from RDW were used to examine the determinants of consumption of IFA, calcium, and weight monitoring throughout pregnancy.

Maternal dietary diversity was assessed using an individual 24‐hr diet recall, recording all foods and beverages consumed in the past 24 hr. These foods were then grouped into 10 categories based on the Minimum Dietary Diversity guidelines for Women (Food and Agriculture Organization and 360, 2016). A diet diversity score was the sum of the 10 food groups and thus ranged from 0 to 10. We also used the cut‐off of five food groups per day as it is recommended for women of reproductive age to achieve their micronutrient needs (Food and Agriculture Organization and 360, 2016).

RDW were asked if they ever consumed IFA or calcium, and the number of IFA and calcium tablets they consumed during their last pregnancy. Current World Health Organization (WHO) guidelines (WHO, 2016) recommend daily IFA supplementation for women throughout pregnancy, and daily calcium supplementation for PW living in areas with low dietary calcium intake. PW should receive these supplements as early as possible, for at least 180 days during pregnancy. Many women often seek ANC late and do not attain these levels of intake. We therefore reported different levels of intake: ever consumed, consumed 45, 90, 100, 135, and 180 tablets. For weight monitoring, RDW were asked if they were ever weighed, and the number of times they were weighed during their last pregnancy.

### 2.3 Independent variables

Independent variables were considered at maternal and household levels. We also examined the potential influence of community and health services factors. Survey questions used to develop these variables are listed in Tables S2 and S3.

At maternal level, we included three behavioural determinants: *knowledge*, *belief*, and *self‐efficacy* related to specific maternal nutrition practices**.** For *knowledge of diet diversity*, PWs were asked about foods that can provide good nutrition for their baby to grow well. Women who reported at least five food groups were given a score of 1 (correct). *Knowledge of IFA and calcium* was assessed based on mothers' responses to several questions: if they ever heard of IFA or calcium supplementation, recommended number of IFA or calcium tablets to be consumed during pregnancy, benefits of IFA or calcium supplements for mother and baby, and about beverages that may decrease iron absorption. Each knowledge question has multiple options, and each was scored 1 if correct and 0 if incorrect. Then, the scores of all relevant question for a given knowledge variable were summed to create a composite score. *Knowledge of weight gain* was assessed based on RDW's knowledge on how much weight a pregnant woman should gain during pregnancy, where a response of 10–12 kg was scored as 1 (correct). *Other behavioural determinants* were measured on a 5‐point Likert scale by asking women the extent to which they agreed or disagreed with statements of *belief and self‐efficacy* related to adopting recommended practices for IFA, calcium, diet diversity, and weight gain. The questions for knowledge, belief, and self‐efficacy were validated in Bangladesh (Nguyen et al., 2017) and were further pilot tested and contextualized for the UP context. For ease of interpretation, the composite scores for knowledge and behavioural determinants were then divided into tertiles (as low, medium, or high knowledge levels) for regression analyses.

#### 2.3.1 Household factors

*Support from family members* was assessed by asking women whether their husbands or mothers/mothers‐in‐law helped to acquire diverse foods or IFA/calcium supplements, reminded them to consume them, monitored their weight, and provided other support during pregnancy. Each statement was given a score of 1 (strongly agree or agree) or 0 (strongly disagree, disagree, neither agree, nor disagree), and the sum of scores was divided into tertiles to obtain high, medium, and low support categories.

#### 2.3.2 Health service factors

Exposure to ANC was measured by asking about the *timing* of the first ANC visit and the *total number of ANC visits*. Timing of first ANC visit was then divided into three periods: early enrolment during the first trimester, intermediate enrolment during the second trimester, or late enrolment during the third trimester of pregnancy. *Maternal exposure to nutrition counselling* was assessed by asking women about various messages they received either during ANC visits or from frontline workers (FLWs) during pregnancy. Women were also asked whether they received *home visits* from FLWs and the total number of visits, and whether they *received IFA/calcium for free*.

#### 2.3.3 Community factors

Perceived *social norms* were measured on a 5‐point Likert scale by asking women the extent to which they agreed or disagreed to what other people in the community expect or think PW should do related to maternal nutrition recommendations. The scales for social norms were constructed by summing score from five statements (each with score of 1 for strongly agree or agree and 0 for other responses), then divided into tertiles to obtain high, medium, and low norm categories.

### 2.4 Control variables

The maternal characteristics that were controlled for in the analyses included religion (Hindu or others), caste (scheduled caste/schedule tribe [SC/ST] or others), education (categorized as illiterate, elementary, middle, and high school or higher), and parity. The household‐level control variables included household food insecurity and socioeconomic status (SES). Household food security index was constructed on the basis of nine items related to the household's experience of food insecurity in the past 30 days (Coates, Swindale, & Bilinsky, 2007), then divided as food secured or in‐secured categories. Household SES was constructed using a principal component analysis of variables on housing conditions and asset holdings; then dividing the first component into tertiles (Filmer & Pritchett, 2001; Vyas & Kumaranayake, 2006).

### 2.5 Statistical analysis

Descriptive analysis was used to report the characteristics of the study population. Bivariate analyses were conducted to test for associations between potential determinants and outcomes of interest. These outcomes were analysed as binary variables (the proportion of PW consumed ≥5 food groups, ever consumed IFA or calcium, and received weight measurement) and count variables (number of food groups or IFA/calcium tablets consumed or number of weight measurements). We then built models to examine multiple determinants at maternal, household, community, and health service levels based on prior conceptual frameworks and key determinants for IFA and dietary diversity from literature review (Table S1). Less is known about calcium supplementation and weight gain monitoring; thus, similar models were developed to identify determinants in this context. Multivariate Poisson regression models were conducted for count outcome variables and logistic regression models for binary outcome variables, adjusting for control variables at maternal and household levels, accounting for variation among Gram Panchayats as a random effect using a cluster sandwich estimator. Prevalence ratio (PR) with its 95% confidence interval was estimated for Poisson regression models, and odds ratio (OR) with its 95% confidence interval was estimated for logistic regression models. Finally, population attributable risk analysis (Newson, 2013) was used to estimate by how much the outcomes can be improved under different scenarios (i.e., exposure to each determinant or combination of determinants), using select modifiable factors that were identified based on the regression results. Among the multiple independent variables described above, we identified factors which are likely “modifiable” by the program as opposed to “nonmodifiable” factors. We consider modifiable factors to be those that may be directly influenced by the program, such as maternal knowledge, beliefs and self‐efficacy, family support, and health services. In contrast, the nonmodifiable factors (such as caste or religion) are the ones that would not change based on program participation. All analysis was done using Stata version 15.

## 3 RESULTS

### 3.1 Sample characteristics

The median age of mothers was 25 years (interquartile range: 22.4–28.0; Table 1). Nearly all women were Hindu (92%), and 41% belonged to SC/ST (SC/ST caste categories). More than a quarter of women were illiterate, and only one third completed high school. Less than 40% women received ANC visits within the first trimester, and only about one fourth of RDWs achieved at least four ANC visits during their last pregnancy. The proportion of PW and RDW visited at home by an FLW during pregnancy were 72% and 83%, respectively. More than two thirds (68%) of PW received counselling on IFA, 12% on calcium, 36% on dietary diversity, and 16% on weight gain. About 60% RDW reported receiving free IFA, and only 14% women received free calcium supplements.

**Table 1:** Sample characteristics

Characteristics	Pregnant women	Recently delivered women
	*n* = 667	*n* = 1,838
	Mean ± *SD* or percent	Mean ± *SD* or percent
Maternal factors		
Maternal age, y	25.05 ± 4.09	25.81 ± 4.29
Religion as Hindus, %	92.05	93.25
Caste category, %		
SC/ST	41.68	41.02
OBC	41.53	44.12
Others	16.79	14.85
Education, %		
No schooling	24.54	28.46
Elementary school	15.55	14.62
Middle school	20.88	22.31
≥High school	39.02	34.61
Parity, *n*	1.20 ± 1.28	2.24 ± 1.32
Vegetarian, %	56.97	51.74
Knowledge scores on IFA,^a^ *n*	2.12 ± 1.45	2.08 ± 1.53
Knowledge scores on calcium,^a^ *n*	0.59 ± 1.15	0.82 ± 1.40
Knowledge on minimum dietary diversity, %	5.55	12.73
Knowledge on weight gain needed during pregnancy, %	4.05	3.75
Belief and self‐efficacy scores,^a^ *n*	7.38 ± 1.02	7.43 ± 1.99
Household factors		
Support from husbands/MMILs score, n	7.21 ± 2.20	6.48 ± 1.58
Household food insecurity, %	16.19	27.09
Household SES index, n	0.00 ± 0.91	0.00 ± 0.95
Community factors		
Social norm scores,^a^ *n*	6.62 ± 1.25	6.50 ± 1.10
Health service factors		
Timing of ANC, %		
Early (<3 months)	39.88	39.55
Intermediate (3–6 months)	35.98	38.47
Late (7–9 months) or no ANC	24.14	21.98
≥4 ANC visits, %	12.14	26.82
Received IFA for free, %	61.62	62.84
Received calcium for free, %	5.10	13.82
Home visit by AWW/ASHA, %	71.51	83.35
Received counselling on IFA, %	67.92	72.25
Received counselling on calcium, %	11.54	20.29
Received counselling on dietary diversity, %	35.53	51.47
Received counselling on weight gain, %	15.89	18.88

Abbreviations: ANC: antenatal care; ASHA: Accredited Social Health Activist; AWW: Anganwadi worker; IFA: iron folic acid; MMIL: mother or mother‐in‐law; OBC: other backward classes; SC: scheduled caste; SES: socioeconomic status; ST: scheduled tribe.

a Scores on knowledge, belief and self‐efficacy, social norms, and household support were scaled and ranged from 0 to 10.

### 3.2 Maternal nutrition practices

**Figure 1:**
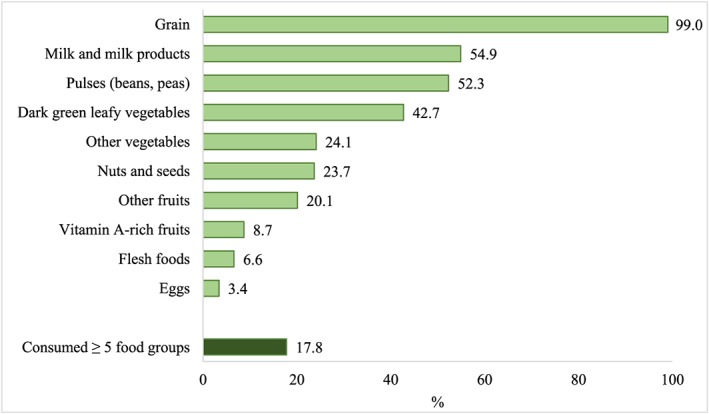
Proportion of pregnant women who consume each of the 10 food groups and ≥ 5 food groups

Although nearly all PW consumed grains, only about half consumed milk and pulses, 43% consumed dark green leafy vegetables, one fifth consumed other vegetables and fruits, and less than 10% consumed vitamin‐A rich fruits, flesh foods, or eggs (Figure 1). On average, PW consumed 3.5 food groups daily and only 18% meet the recommendation of at least five food groups.

**Figure 2:**
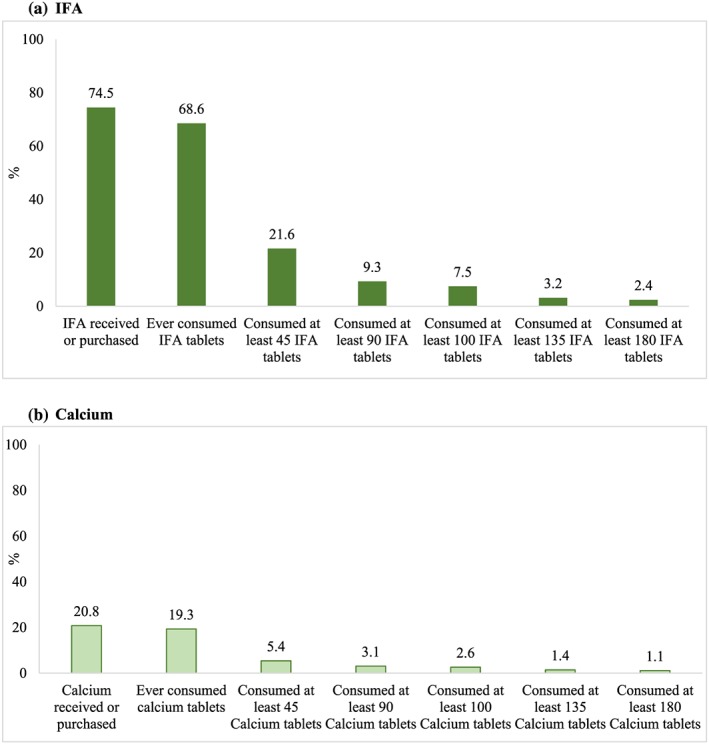
Receipt and consumption of iron folic acid (IFA) (a) and calcium (b) during the last pregnancy among recently delivery women. 1. Percentages reported are among all women. 2. Among all women, mean IFA and calcium consumption during pregnancy were 28.3 and 8.2, respectively

Overall, about three quarters of RDW received or purchased IFA, and 69% of RDW ever consumed IFA during pregnancy (Figure 2). However, among all women, only 2% met the recommendation of consuming at least 180 IFA tablets during pregnancy. The proportion of IFA consumption was also low when using different cut‐offs of 135 tablets (3%), 100 tablets (8%), or 45 tablets (22%). Receipt and consumption of calcium were even lower, at ~20%, and only 1% and 3% RDW consumed 180 and 100 tablets, respectively. On average, among all participants, women only consumed 28 IFA and 8 calcium tablets during pregnancy. Among women who had ever consumed supplements, the average consumption was 41–43 tablets, respectively. Around 63% of women were weighed at least once during their pregnancy and only 17% of women were weighed at least 3 times, with an average number of 1.3 times during their last pregnancy (results not shown).

### 3.3 Determinants of diet diversity

Maternal knowledge on diet diversity during pregnancy was strongly associated with the proportion consuming five food groups and number of food groups consumed (Table 2). Compared with women with low knowledge, those with high knowledge consumed more food groups (PR = 1.19 times) and were 2.2 times more likely to consume ≥5 food groups. Women who received dietary counselling were approximately twice more likely to consume at least five food groups. Maternal education and household SES were also positively associated with dietary diversity; women with middle or high school education consumed 12% more food groups compared with those with no schooling, and women from higher wealth groups consumed 10% more food groups compared with those from lower wealth groups. Women belonging to backward classes (other backward classes) consumed fewer food groups (PR = 0.91) than higher caste group.

**Table 2:** Determinants associated with diet diversity among pregnant women

Determinant factors	Consumed at least five food groups		Number of food group consumed	
	*n* = 667		*n* = 667	
	OR	95% CI	PR	95% CI
Maternal factors				
Knowledge on dietary diversity	2.15+	0.88,5.24	1.19**	1.07,1.32
Belief and self‐efficacy				
Medium	0.63	0.32,1.24	0.98	0.90,1.07
High	0.81	0.45,1.47	1.03	0.94,1.11
Household factors				
Support from family members (low as ref)				
Medium	0.76	0.43,1.34	1.01	0.94,1.10
High	1.22	0.74,2.02	1.05	0.97,1.13
Community factors				
Social norms (low as ref)				
Medium	0.93	0.45,1.92	1.01	0.92,1.11
High	1.42	0.72,2.81	1.04	0.94,1.15
Health service factors				
Timing of ANC (late as ref)				
Intermediate (3–6 months)	0.88	0.46,1.69	0.97	0.89,1.06
Early (<3 months)	1.37	0.69,2.71	1.02	0.93,1.11
≥ 4ANC	0.83	0.43,1.60	1.05	0.97,1.14
Home visited by FlW	0.72	0.43,1.21	0.98	0.92,1.06
Received dietary diversity counselling	1.91*	1.16,3.15	1.09*	1.01,1.17
Control variables				
Religion as Hindus	0.56	0.24,1.28	0.92	0.82,1.04
Caste category (others as ref)				
SC	0.51+	0.26,1.03	0.93	0.85,1.02
OBC	0.53*	0.30,0.93	0.91*	0.84,0.98
Education (illiterate as ref)				
Elementary school	0.70	0.30,1.62	1.07	0.95,1.2
Middle school	1.16	0.54,2.48	1.12*	1.00,1.25
High school or higher	1.08	0.51,2.29	1.12*	1.00,1.25
Parity (0 as ref)				
1	0.97	0.56,1.69	0.99	0.91,1.07
2	0.65	0.33,1.26	0.89**	0.82,0.97
≥3	0.67	0.30,1.52	0.94	0.84,1.06
Number of months in pregnancy	1.25**	1.06,1.47	1.02	0.99,1.04
Woman as a vegetarian	0.77	0.46,1.30	1.01	0.95,1.08
Household SES (low as ref)				
Medium	1.02	0.55,1.90	0.99	0.92,1.08
High	1.68	0.87,3.25	1.10*	1.00,1.21
Food security	1.16	0.57,2.35	0.99	0.90,1.10

Abbreviations: CI: confidence interval; FLW: frontline worker; IFA: iron folic acid; OBC: other backward classes; OR: odds ratio; PR: prevalence ratio; SC: scheduled caste; SES: socioeconomic status.

+ *p* < .10.

* *p* < .05.

** *p* < .01.

### 3.4 Determinants of IFA and calcium supplement consumption

Maternal knowledge of IFA and calcium supplements was strongly associated with ever consuming and number of IFA (Table 3) and calcium supplements (Table 4). Compared with those with low knowledge score, women with high knowledge scores were more likely to consume IFA (OR = 2.3) or calcium (OR = 11.7) and consumed more IFA (1.8 times) or calcium tablets (6.0 times). High levels of belief and self‐efficacy among women were also associated with increased odds of consuming IFA (OR = 2.0) and calcium (OR = 4.6) supplements.

**Table 3:** Determinants associated with consumption of IFA tablets among recently delivered women

Determinant factors	Ever consumed IFA		Number of IFA consumed	
	*n* = 1,838		*n* = 1,838	
	OR	95% CI	PR	95% CI
Maternal factors				
Knowledge on IFA (low as ref)				
Medium	1.89**	1.26,2.84	1.40**	1.16,1.70
High	2.31***	1.46,3.66	1.80***	1.46,2.21
Belief and self‐efficacy				
Medium	1.64*	1.08,2.49	1.45***	1.24,1.70
High	1.95**	1.19,3.18	1.46**	1.21,1.76
Household factors				
Support from family members (low as ref)				
Medium	1.28	0.85,1.91	1.12+	0.98,1.28
High	1.51+	0.97,2.35	1.19*	1.02,1.39
Community factors				
Social norms (low as ref)				
Medium	1.06	0.71,1.58	1.20*	1.03,1.39
High	1.15	0.75,1.75	1.32***	1.13,1.54
Health service factors				
Timing of ANC (late as ref)				
Intermediate (3–6 months)	1.08	0.69,1.68	1.01	0.83,1.23
Early (<3 months)	1.60*	1.03,2.47	0.98	0.80,1.19
***≥ 4ANC***	1.17	0.79,1.73	1.26**	1.08,1.47
Received IFA free	14.00***	9.34,20.99	1.11	0.92,1.34
Home visited by FLW	1.29	0.86,1.92	0.86	0.71,1.03
Receive IFA counselling	10.37***	7.45,14.43	3.56***	2.45,5.16
Control variables				
Religion as Hindus	1.07	0.64,1.79	1.06	0.82,1.36
Caste category (others as ref)				
SC	0.92	0.57,1.50	0.81+	0.66,1.00
OBC	0.86	0.51,1.45	0.91	0.76,1.09
Education (illiterate as ref)				
Elementary school	0.94	0.56,1.58	0.97	0.77,1.22
Middle school	1.23	0.79,1.92	1.11	0.92,1.35
High school or higher	1.57+	0.96,2.57	1.15	0.91,1.45
Parity (1 as ref)				
2	0.91	0.62,1.33	0.93	0.79,1.10
3	0.91	0.56,1.49	0.94	0.78,1.12
≥4	0.68	0.40,1.15	0.83+	0.67,1.02
Household SES (low as ref)				
Medium	0.61*	0.41,0.90	0.91	0.78,1.06
High	0.76	0.47,1.24	1.03	0.86,1.23
Food security	1.02	0.68,1.55	1.07	0.91,1.27

Abbreviations: CI: confidence interval; FLW: frontline worker; IFA: iron folic acid; OBC: other backward classes; OR: odds ratio; PR: prevalence ratio; SC: scheduled caste; SES: socioeconomic status.

+ *p* < .01.

* *p* < .05.

** *p* < .01.

*** *p* < .001.

**Table 4:** Determinants associated with consumption of calcium tablets among recently delivered women

Determinant factors	Ever consumed calcium		Number of calcium consumed	
	*n* = 1,838		*n* = 1,838	
	OR	95% CI	PR	95% CI
Maternal factors				
Knowledge on calcium (low as ref)				
Medium	8.26***	4.46,15.27	3.13***	1.74,5.61
High	11.69***	5.97,22.86	6.04***	3.32,11.01
Belief and self‐efficacy				
Medium	1.42	0.65,3.09	2.56***	1.58,4.14
High	4.63***	2.03,10.52	2.77***	1.68,4.57
Household factors				
Support from family members (low as ref)				
Medium	1.03	0.47,2.29	1.06	0.66,1.68
High	2.06*	1.02,4.17	1.57*	1.02,2.42
Community factors				
Social norms (low as ref)				
Medium	1.41	0.74,2.67	1.74**	1.16,2.63
High	1.15	0.57,2.31	1.82**	1.17,2.83
Health service factors				
Timing of ANC (late as ref)				
Intermediate (3–6 months)	0.93	0.44,1.97	0.89	0.52,1.53
Early (<3 months)	1.36	0.65,2.85	0.92	0.54,1.59
≥4ANC	2.20**	1.25,3.87	1.82***	1.30,2.55
Received calcium free	58.63***	24.83,138.44	1.62**	1.18,2.22
Home visited by FlW	0.96	0.45,2.05	0.97	0.94,1.01
Receive calcium counselling	13.47***	7.85,23.12	2.66**	1.48,4.77
Control variables				
Religion as Hindus	0.91	0.33,2.50	0.98	0.53,1.79
Caste category (others as ref)				
SC	0.92	0.50,1.69	0.56**	0.37,0.84
OBC	0.77	0.40,1.48	0.66*	0.46,0.95
Education (illiterate as ref)				
Elementary school	0.71	0.30,1.65	1.19	0.68,2.08
Middle school	0.77	0.37,1.60	1.38	0.89,2.14
High school or higher	1.66	0.86,3.24	1.68*	1.12,2.53
Parity (1 as ref)				
2	1.22	0.66,2.25	0.87	0.62,1.23
3	1.67	0.81,3.42	1.48	0.89,2.48
≥4	1.58	0.60,4.16	0.83	0.45,1.52
Household SES (low as ref)				
Medium	1.39	0.69,2.79	1.06	0.70,1.61
High	1.82	0.77,4.30	1.45	0.90,2.33
Food security	0.57*	0.33,0.98	1.10	0.81,1.51

Abbreviations: CI: confidence interval; FLW: frontline worker; IFA: iron folic acid; OBC: other backward classes; OR: odds ratio; PR: prevalence ratio; SC: scheduled caste; SES: socioeconomic status.

* *p* < .05.

** *p* < 0.01.

*** *p* < .001.

Women with high family support reported increased consumption of IFA (OR = 1.5) and calcium (OR = 2.1). The influence of social norms was significant for both IFA and calcium consumption; with a 32% and 82% increase in number of the IFA and calcium tablets consumed among women with high social norms, respectively.

Early ANC was significantly associated with ever consuming IFA (OR = 1.6), but not with the number of IFA or calcium tablets consumption. Women who received at least four ANC check‐ups had a greater likelihood of consuming IFA and calcium (26% more IFA tablets and 82% more calcium tablets). Compared with those who had to buy supplements, those who received free IFA were 14 times more likely to consume, and those who received free calcium were 59 times more likely to consume them. Receiving counselling was associated with higher likelihood of ever consuming IFA and calcium (OR = 10.4 and 13.5, respectively) and number of tablets consumed (3.6 and 2.7 times, respectively). Women's background characteristics including caste and education were associated with number of calcium supplements consumed. Compared with those with no schooling, women who completed high school consumed 68% additional calcium tablets. Conversely, those belonging to SC or other backward classes consumed fewer calcium tablets (PR = 0.56 and 0.66, respectively).

### 3.5 Determinants of weight monitoring

Correct knowledge of adequate weight gain among women was associated with increased odds of having their weight monitored with greater frequency (1.3 times; Table 5). Compared with women with low family support, those who received high support from family members were 1.4 times more likely to be weighed during pregnancy.

**Table 5:** Determinants associated with weight monitoring among recently delivered women

Determinant factors	Ever weighed		Number of times weighed	
	*n* = 1,838		*n* = 1,838	
	OR	95% CI	PR	95% CI
Maternal factors				
Knowledge on weight gain	1.39	0.80,2.41	1.34**	1.11,1.63
Household factors				
Support from family members (low as ref)				
Medium	1.37*	1.07,1.75	1.16*	1.03,1.30
High	1.36*	1.03,1.79	1.23**	1.09,1.39
Health service factors				
Timing of ANC (late as ref)				
Intermediate (3–6 months)	2.57***	1.95,3.39	1.43***	1.20,1.71
Early (<3 months)	2.98***	2.24,3.97	1.68***	1.40,2.01
≥4ANC	1.56**	1.11,2.17	1.47***	1.31,1.64
Home visited by FLW	1.33*	1.02,1.75	0.95	0.81,1.12
Received counselling on weight gain	2.19***	1.62,2.95	1.31***	1.16,1.47
Control variables				
Religion as Hindus	1.76*	1.10,2.83	1.24*	1.01,1.54
Caste category (others as ref)				
SC	0.91	0.62,1.35	0.85*	0.73,1.00
OBC	1.14	0.78,1.66	0.87+	0.75,1.01
Education (illiterate as ref)				
Elementary school	1.09	0.80,1.50	1.10	0.93,1.31
Middle school	1.36*	1.01,1.83	1.26**	1.09,1.45
High school or higher	1.26	0.91,1.75	1.22*	1.05,1.43
Parity (1 as ref)				
2	1.14	0.86,1.51	0.99	0.87,1.12
3	1.13	0.80,1.60	1.03	0.87,1.21
≥4	1.21	0.90,1.63	1.07	0.92,1.23
Household SES (low as ref)				
Medium	0.93	0.71,1.20	0.96	0.84,1.08
High	1.18	0.87,1.60	1.08	0.95,1.24
Food security	0.98	0.77,1.26	1.01	0.91,1.13

Abbreviations: CI: confidence interval; FLW: frontline worker; IFA: iron folic acid; OBC: other backward classes; OR: odds ratio; PR: prevalence ratio; SC: scheduled caste; SES: socioeconomic status.

* *p* < .05.

** *p* < .01.

*** *p* < .001.

Receiving early ANC was significantly associated with ever being weighed and number of times being weighed. Compared with women who received late ANC, those who received it within their first trimester were 3 times more likely to be weighed and were weighed 68% times more. Receiving four or more ANC visits were also associated with increased odds of being weighed during pregnancy (OR = 1.6) and were weighed 47% additional times. Receiving a home visit by a FLW was associated with increased odds of being weighed (OR 1.3) and receiving counselling on weight gain during pregnancy was associated with increased both odds of being weighted (OR = 2.2, respectively) as well as an increased frequency of weight monitoring (31%). Women who completed middle or high school were weighed with higher frequency (22–36% additional number of times during pregnancy), and those who belonged to SC groups were weighed with lower frequency (PR = 0.85).

### 3.6 Population attributable risk estimation

**Figure 3:**
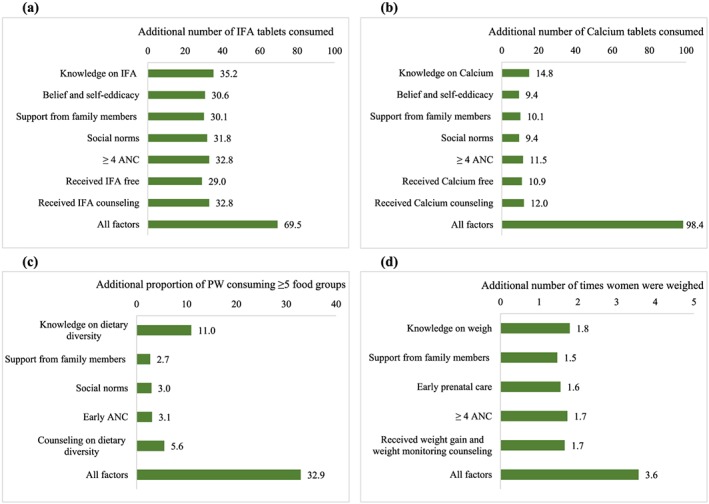
Population attributable risk estimations of the influence of select modifiable factors on maternal nutrition practices: (a) additional number of iron folic acid (IFA) tablets consumed, (b) additional number of Calcium tablets consumed, (c) additional proportion of pregnant women (PW) consuming ≥5 food groups, and (d) additional number of times women were weighed

The variables selected in the final regression models from Tables 2, 3, 4, 5 addressed the question of which factors significantly explained consumption of supplements and dietary diversity, as well as weight monitoring. Selected modifiable factors from these models were then used in population attributable risk analysis to estimate how much the outcomes could be improved if the new interventions being tested by A&T could change these determinants to the highest level (best case scenarios/optimal program implementation) compared with the current baseline scenarios. Under combined conditions of good knowledge, high support from family members, positive social norms, and early ANC visits, we can expect a 33% increase in the proportion of PW consuming diverse diets (Figure 3). Given that 18% PW currently consume ≥5 food groups, this would result in a total of 51% PW following recommendations on diet diversity.

The population attributable risk analyses also indicated that under combined conditions of high knowledge, beliefs/self‐efficacy, family support, social norms, and optimal access to health services, women would consume additional 70 IFA and 98 calcium tablets during pregnancy. Among the individual factors, knowledge on IFA and calcium contributes to the highest increase in number of tablets (35 IFA and 15 calcium, respectively). Improvements in all factors would add to the current mean consumption (28 IFA and 8 calcium tablets), resulting in a total of 98 IFA and 106 calcium tablets consumed, still far from the recommendations for IFA and calcium supplementation during pregnancy. These changes in determinants also lead to an additional 3.6 times women's weight would be monitored, resulting in women being weighed 4.9 times during pregnancy (current 1.3 times).

## 4 DISCUSSION

Our study provides a comprehensive assessment of the multiple determinants (individual, family, community, and health service factors) associated with key maternal nutrition practices in Uttar Pradesh. We found each maternal nutrition practice to be influenced by a varying set of determinants. Among the multiple factors, maternal behavioural characteristics (such as knowledge, beliefs, and self‐efficacy), followed by support from family members, community factors, and adequate health services access, were significantly associated with key maternal nutrition practices. In addition, we quantified what can be achieved through a set of strategies to optimize practices.

To model potential optimal program implementation characterized by high‐quality behaviour change communication and access to health services, we estimated the program impact if all women were able to achieve high levels of knowledge, beliefs/self‐efficacy, family support, positive social norms, and use of health services. Based on this, we estimated a 33% increase in diet diversity, increased consumption of an additional 70 IFA and 98 calcium tablets, and 3.6 times increase in weight monitoring during pregnancy. Although these finding are encouraging, delivering the optimal program will be considerably challenging, requiring substantial investments in system strengthening and closing supply chain gaps. Even if optimal implementation is achieved, it is important to note that resulting levels of maternal nutrition practices would still be far from meeting WHO recommendations.

Maternal diet diversity is a complex outcome and was found to be associated with maternal knowledge and receipt of counselling. Collectively improvement in these factors would result in about half of women meeting recommendations on diet diversity. Additional nonmodifiable factors associated with dietary diversity included caste, education, and economic status. Our results are consistent with previous literature wherein maternal knowledge and socio‐economic status were found to be associated with dietary diversity (de Castro et al., 2016; Doyle, Borrmann, Grosser, Razum, & Spallek, 2017; Rosen et al., 2018; Shamim et al., 2016). Women belonging to higher caste and income groups were more likely to have a diverse diet, suggesting a resource constraint in adoption of diet recommendations beyond improved knowledge and counselling services. Therefore, food transfers (already part of the Integrated Child Development Scheme services in India) or cash transfers could be one of the mechanisms to improve household diet. Evidence suggests that unconditional and conditional cash transfers improve household food consumption and dietary diversity (Alderman, 2016), although it is unclear if similar improvements can be achieved in maternal diet diversity.

IFA consumption was strongly associated with maternal knowledge, followed by counselling, high beliefs/self‐efficacy, positive social norms, ANC visits, and support from family members. Under optimal program implementation, these factors combined could result in an average consumption of 100 IFA tablets during pregnancy. Although this is below the recommended levels, it is substantially better than the current situation (approximately 40 IFA tablets are consumed during pregnancy). Our findings align well with existing literature from Bihar, Pakistan, Indonesia, and Kenya related to associations of maternal knowledge and family support in ensuring sustained IFA adherence (Birhanu et al., 2018; Wiradnyani, Khusun, Achadi, Ocviyanti, & Shankar, 2016). Prior research also highlights the role of education and wealth for compliance to IFA recommendations (Nisar, Dibley, & Mir, 2014; Wendt et al., 2015). On the supply side, provision of supplements, counselling, and prenatal care services appear to be critical. Wendt et al. (2015) found that women in Bihar who received any IFA were more likely to consume for 90 or more days if they attended four or more ANC. Important gaps in IFA supply include stock‐outs, lack of personnel, procurement, storage, and unsystematic distribution (Wendt et al., 2018). Closing similar supply chain gaps will likely improve consumption among women in UP as well. Our findings also reinforce the role of counselling services and ANC contacts in improving IFA consumption (Kamau, Mirie, & Kimani, 2018; Nisar et al., 2014; Sununtnasuk, D'Agostino, & Fiedler, 2016; Wiradnyani et al., 2016).

In comparison to IFA compliance, much less is known globally about barriers and facilitators related to calcium consumption. Qualitative findings from Ethiopia and Kenya suggest that awareness of risks and benefits of calcium along with family support were critical in attaining sustained adherence (Birhanu et al., 2018; Martin et al., 2017; Martin et al., 2017). Lack of awareness of preeclampsia/eclampsia and apprehensions around coconsumption of IFA and calcium were identified as key demand‐side barriers in these contexts (Birhanu et al., 2018), which provides pertinent guidance for structuring counselling messages.. Similar to IFA, a key limiting factor will be availability and accessibility of calcium supplements. Experience from Nepal reiterates the need for strengthening supply‐side factors including provision of complete ANC services and uninterrupted provision of supplements, which were associated with consumption of full‐course of calcium among PW who received them (Thapa et al., 2016). Consistent with literature, our study shows that maternal knowledge, receiving counselling on calcium supplementation, beliefs and self‐efficacy, ANC visits, and positive social norms were associated with increased consumption of calcium. Under optimal program conditions, we estimate an average consumption of 90 calcium tablets. Again, this is below the recommended levels, and it is more than double the current minimal calcium consumption.

The new WHO guidelines recommended counselling about healthy eating and keeping physically active during pregnancy for PW to stay healthy and to prevent excessive weight gain during pregnancy (WHO, 2016). On the other extreme, many PW in developing countries are struggling to achieve adequate gestational weight gain. Therefore, regular monitoring of weight gain during pregnancy is critical. To our knowledge, there is no literature on the determinants associated with weight gain monitoring during pregnancy. In our study, early and frequent ANC visits, together with counselling on weight gain, were found to be associated with both higher odds of being weighed and additional weight monitoring.

Under India's current policy framework, the Pradhan Mantri Surakshit Matritva Abhiyan aims at ensuring ANC check‐ups on a fixed given day (usually ninth of each month) during the second or third trimester of pregnancy by a doctor at a government health facility (Ministry of Health & Family Welfare, 2016). This offers an opportunity for improving ANC, counselling on micronutrient supplementation, and for regular weight monitoring. Although 40% of women in our study areas reported using public‐sector facilities for ANC, between 11 and 15% women used private facilities (analysis from NFHS‐4, unpublished), suggesting that it is important to examine the quality of ANC in private facilities to ensure that all the recommended interventions are being delivered. In addition, the maternity benefits scheme (Ministry of Women and Child Development, 2017) aims at improving the health seeking behaviour among pregnant and lactating women. Furthermore, there are intense efforts to bring about changes in social normative behaviours through community mobilization activities and mass media campaigns (Ministry of Women and Child Development, 2018). The current study findings emphasize the need for focused implementation of these policy efforts to ensure adequate number of contacts, adequate supply of micronutrient supplements, and appropriate behaviour change counselling.

Our study provides comprehensive information on the multiple factors related to adoption of maternal nutrition practices at multiple levels including individual, household, community, and health services. Importantly, we find each specific maternal nutrition practices to be influenced by differing sets of determinants. Given that diverse diets, IFA and calcium supplements, and weight monitoring are promoted and delivered through a common package, a nuanced understanding of the drivers for each specific practice can help improving access to the full package. Through modelling approaches, the effects of individual and combined factors help identify and highlight improvements that may be achieved by the program. In particular, our findings provide new evidence on determinants of calcium consumption and weight monitoring during pregnancy for India, topics with a very limited literature both in India and elsewhere.

We acknowledge some methodological limitations of our analyses. First, the cross‐sectional design limits conclusions about causality. Second, maternal nutrition practices are self‐reported by women and are subject to potential recall bias and social desirability bias. Estimates for consumption of IFA and calcium are contingent on consistent supply chains across the program duration. However, program efforts are in place for strengthening the procurement and management of these supplements and will be closely monitored.

## 5 CONCLUSION

Our study provides novel findings on the multiple determinants of specific maternal nutrition practices in Uttar Pradesh, India. Although the current situation is bleak, many of the key factors associated with maternal dietary diversity, IFA/calcium consumption, and weight gain monitoring are modifiable. Under optimal program implementation that explicitly tackles the specific determinants of each maternal nutrition practice, we could expect half of the women to achieve adequate diet diversity, consume an average of 100 IFA and 90 calcium tablets, and women to be weighed 4.4 times during their pregnancy. Strengthening existing program operations to improve the supply of interventions and to create adequate demand through behaviour change communication has the potential to result in large improvements in maternal nutrition practices from their baseline levels. At the same time, our findings highlight that these efforts will not be sufficient to meet WHO‐recommended levels without investing in resources, which enable improvements in socio‐economic conditions, such as education, income, or employment generating activities to support and sustain behaviour change.

## CONFLICTS OF INTEREST

The authors declare that they have no conflicts of interest.

## CONTRIBUTIONS

PHN conceived the paper, conducted the data analysis, drafted the manuscript, consolidated comments from all coauthors, and revised and finalize the paper. SK was responsible for field work coordination, data analyses, literature review and drafted some parts of the manuscript. RA and MY interpreted the data, provided inputs for introduction/discussion, and reviewed the manuscript. LMT conducted the data analyses and prepared the tables and figures. SG, RA, and JE interpreted the data and its implications and provided overall comments. SP provided inputs for data analysis and contributed to draft sections (e.g., sampling and measurements), draft edit, and reviewed the manuscript. PM interpreted the data, provided inputs for introduction/discussion, and reviewed the manuscript. All authors read and approved the final submitted manuscript.

## Supporting information

Table S1: Review of determinants of maternal nutrition practices
Table S2: Questions used to create knowledge, belief and self‐efficacy, social norms, and supports from husbands, mothers and mothers in law
Table S3: Counselling messages received for specific maternal nutrition practices
