# Supplementary material for: Maternal nutrition practices in Uttar Pradesh, India: Role of key influential demand and supply factors
Source: Matern Child Nutr. 2019 Jun 18;15(4):e12839. doi: 10.1111/mcn.12839 (PMC6852235; doi:10.1111/mcn.12839)
Supplement: Supplementary file 1 — Table S1: Review of determinants of maternal nutrition practices Table S2: Questions used to create knowledge, belief and self‐efficacy, social norms, and supports from husbands, mothers and mothers in law Table S3: Counselling messages received for specific maternal nutrition practices [file MCN-15-e12839-s001.docx]

**Supplementary Table 1: Review of determinants of maternal nutrition practices**

| **Author/year** | **Country** | **Outcomes** | **Determinants** | |
| --- | --- | --- | --- | --- |
|  |  |  | **Demand side** | **Supply side** |
| Birhanu *et al.*, 2018 | Ethiopia | IFA supplement consumption | - Early and regular ANC attendance - Knowledge, motivation and willingness to take IFA | - Supply of supplements - Trained and motivated health staff |
| Wendt *et al.*, 2018 | India | IFA supplement supply change |  | - IFA forecasting, procurement, storage,   disposal   - Lack of personnel, and few training opportunities |
| Kamau *et al.*, 2018 | Kenya | IFA supplement consumption | - Sociodemographic characteristics (age, education and occupation - Health care related factors (IFA advice, parity, gravidity and gestation) - Knowledge on IFA |  |
| Wiradnyani *et al.*, 2016 | Indonesia | IFA supplement consumption | - Sociodemographic factors   (wealth index, residence, age, pregnancy order, education)   - Health service access (perceived problems, frequency of ANC visits) - Knowledge on pregnancy related risks - Family and husband support |  |
| Sununtnasuk *et al.*, 2016 | DHS data of 22 countries | IFA distribution and consumption | - Cultural, economic, geographical and   social factors   - Care seeking behavior | - ANC program in health care systems (staff, training, manager, finance) - Adequate supply: logistic system - Providers’ motivation |
| Wendt *et al.*, 2015 | India | IFA receipt and consumption | - Individual level: early ANC, frequency of ANC, and ANC quality | - Facility-level factors: Village health day and   primary health center village monitoring, staff characteristics, and health center infrastructure |
| Nisar *et al.*, 2014 | Pakistan | IFA supplement consumption | - Sociodemographic factors: age, education, occupation, parity, number of living children, residence, SES index - Healthcare utilization factors | - Lady health workers program |
| Martin *et al.*, 2017a | Kennya | Calcium supplement consumption | - Awareness - ANC attendance - Link to IFA supplement |  |
| Martin *et al.*, 2017b | Ethiopia and  Kenya | Calcium supplement consumption | - Acceptability - Adherence - Link to IFA supplements |  |
| Thapa *et al.*, 2016 | Nepal | Calcium supplement | - Evaluate coverage, compliance, acceptability and feasibility of the intervention |  |
| Rosen *et al.*, 2018 | Niger | Dietary practices | - Perception of ideal diet - Barriers: food cost - Dietary responses related to pregnancy illnesses - Changes in perceptions from early to late pregnancy. |  |
| Doyle *et al.*, 2017 | Review of 12 studies | Dietary patterns and diet quality | - Environmental factor: physical, sociocultural, economic/   financial and political factors.   - Sociodemographic factors: age, education, employment, ethnicity - Individual responses - Perinatal period - Pregnancy related factors. |  |
| de Castro *et al.*, 2016 | Brazil | Dietary pattern | - Sociodemographic factors: schooling, parity, marital status, age, skin color and monthly per capita income |  |
| Shamim *et al.*, 2016 | Bangladesh | Diet quality | - Sociodemographic factors: age, education, husband’s occupation, SES - Food security |  |
| Nguyen *et al.*, 2017 | Bangladesh | - IFA and calcium consumption - Dietary diversity - Weight monitoring | - Maternal factors: knowledge, belief, self-efficacy, age, education, parity - Household factors: husband and family support. SES and food security - Health service factors: early ANC, number of ANC visits | - Supply of IFA and calcium |

**Supplementary Table 2: Questions used to create knowledge, belief and self-efficacy, social norms, and supports from husbands, mothers and mothers in law**

| **Questions related to knowledge** |
| --- |
| ***Maternal dietary diversity*** |
| How should a pregnant/lactating woman eat in comparison with a non-pregnant woman to provide good nutrition to her baby and help him grow? |
| ***IFA Knowledge*** |
| Have you ever heard about IFA tablets? |
| How many IFA tablets do you think a pregnant woman should take *in one month*? |
| How many IFA tablets do you think a pregnant woman should take *during pregnancy*? |
| Why do you think a pregnant woman should take iron folic tables? |
| How long after birth should a woman continue taking IFA tablets |
| Some beverages decrease iron absorption when taken with meals. Which ones? |
| ***Calcium knowledge*** |
| Have you ever heard about calcium tablets? |
| How many calcium tables do you think a pregnant woman should take in one month? |
| How many calcium tablets do you think a pregnant woman should take during pregnancy? |
| Why do you think a pregnant woman should take calcium tables? |
| For how long after birth a woman should continue taking calcium? |
| ***Weight gain knowledge*** |
| How much weight should a pregnant woman gain during pregnancy? |
| **Questions related to belief and self-efficacy *(****Please tell me whether you strongly disagree, disagree, neither agree nor disagree, agree or strongly agree with each of the following statements)* |
| My consuming of IFA every day during pregnancy is important for my health and my unborn child |
| My consuming of calcium every day during pregnancy is important for my health and my unborn child |
| My consuming right types and amount of food during pregnancy is extremely important for my health and my unborn child’s health |
| My consuming right types and amount of food during pregnancy can save cost due to doctors and medicine |
| I can follow the recommendations of 5 varieties of food and adequate amounts of food to be consumed along with roti/rice during pregnancy |
| It is too costly to obtain the recommended types and amounts of foods for my consumption during pregnancy |
| I can follow the recommendations of taking IFA every day during pregnancy |
| I can follow the recommendations of taking Calcium everyday |
| **Questions related to support from husband and mothers/ mother in laws *(****Please tell me whether you strongly disagree, disagree, neither agree nor disagree, agree or strongly agree with each of the following statements)* |
| My husband purchased diversified nutritious foods and ensures that I have these foods available for cooking |
| My husband reminded and encouraged me to consume the recommended quantity of diversified foods daily |
| My husband ensured that there are enough tablets of IFA at home and remind me to consume them |
| My husband ensured that there are enough Calcium tablets at home and remind me to consume them |
| My husband helps me to take rest for 2 hours during the day |
| My husband and family members made me work which included heavy lifting during pregnancy |
| My husband reminded me to have my weight checked regularly and recorded in the MCP card |
| My mother/mother- in-law ensured that diverse nutrient rich food items are bought and cooked for my consumption during my pregnancy |
| My mother/mother- in-law reminded me to take IFA tablets daily |
| My mother/mother- in-law reminded me to take calcium tablets daily |
| My mother/mother- in-law reminded me to have my weight checked regularly and record the weight in the MCP card |
| **Questions related to social norms *(****Please tell me whether you strongly disagree, disagree, neither agree nor disagree, agree or strongly agree with each of the following statements)* |
| In my family and community, we/people expect pregnant women to consume five varieties and larger quantity of food to get enough energy and nutrition during pregnancy |
| Most people who are important to me (e.g. family members, friends…) think that a pregnant woman should not eat too much to avoid difficult labor due to large baby |
| In my family and community, pregnant women are expected to avoid certain kinds of foods (meat, fish, papaya, jackfruit, milk etc) because it will harm the mother and/or baby |
| Most people who are important to me (e.g. family members, friends…) think that a pregnant woman should take IFA every day during pregnancy |
| Most people who are important to me (e.g. family members, friends…) think that a pregnant woman should take calcium every day during pregnancy |

**Supplementary Table 3: Counseling messages received for specific maternal nutrition practices**

| Ever received counseling on dietary diversity |
| --- |
| Messages on dietary diversity counseling: |
| Eat five different types of food in addition to roti/rice |
| Consume thick daal (pulses & lentils) everyday |
| Consume Milk/ Milk Product (curd, buttermilk, paneer) daily |
| Consume Dark Green leafy vegetable daily |
| Consume Yellow/ Orange fruit and vegetable daily |
| Consume Egg daily, if acceptable |
| Consume Fish/Meat daily, if non-vegetarian |
| Increase the quantity of milk and milk products if you don’t eat eggs or meat |
| Take nutritious snacks 2-3 times/day |
| Consume extra food with every meal |
| Why different varieties are required |
| Consume jaggery, channa |
| Ever received counseling on IFA |
| Messages on IFA counseling: |
| Take 1 tablet daily during pregnancy |
| Take 180 IFA tablets during pregnancy |
| Continue to take 1 tablet/day till 6 months postpartum/ during lactation |
| Take IFA with water or lemon water |
| Do not take IFA with tea or milk |
| Take IFA at night before bed time/after dinner |
| Do not take IFA and calcium together |
| Do not take IFA tablet on an empty stomach |
| How to remind herself or have family member’s support to take one tablet daily |
| IFA prevents anemia |
| IFA reduces risk of low birth weight baby |
| IFA reduces risk of maternal death due to hemorrhage |
| IFA ensures the best development of the child |
| IFA reduces complication during pregnancy and birth |
| Information related to side effects |
| Increase intake of fruits and vegetable to avoid constipation |
| Consume IFA tablets |
| Ever received counseling on Calcium |
| Messages on Calcium counseling: |
| Take 1- 2 tablets daily during pregnancy |
| Take 360 calcium tablets during pregnancy |
| Continue to take 1-2 tablets/day till 6 months postpartum |
| Do not take IFA and calcium tablets together |
| Do not take calcium tablet on an empty stomach |
| Take the first calcium tablet after breakfast and the second tablet with lunch |
| Calcium helps in the development of bone and teeth of the baby |
| Calcium reduce risk of high blood pressure, swelling of body, headache, vomiting, fits (convulsions). |
| How to remind herself or have family member’s support to take one tablet daily |
| Consume calcium tablets |
| Ever received counseling on Weight gain and weight monitoring |
| Messages on Weight gain and weight monitoring counseling: |
| Weigh yourself regularly |
| Record weight in MCP card |
| Women should gain 1.5-2 kg/month from the fourth month of pregnancy |
| A woman should gain 10-12kg weight during pregnancy |
| Gaining weight indicates proper growth of the fetus |
| Gaining weight indicates mother is taking adequate food |
